# Socio-Psychological Factors Driving Adult Vaccination: A Qualitative Study

**DOI:** 10.1371/journal.pone.0113503

**Published:** 2014-12-09

**Authors:** Ana Wheelock, Anam Parand, Bruno Rigole, Angus Thomson, Marisa Miraldo, Charles Vincent, Nick Sevdalis

**Affiliations:** 1 Faculty of Medicine, Imperial College London, London, United Kingdom; 2 Sanofi Pasteur, Lyon, France; 3 Department of Experimental Psychology, University of Oxford, Oxford, United Kingdom; 4 Imperial College Business School, London, United Kingdom; University of California Riverside, United States of America

## Abstract

**Background:**

While immunization is one of the most effective and successful public health interventions, there are still up to 30,000 deaths in major developed economies each year due to vaccine-preventable diseases, almost all in adults. In the UK, despite comparatively high vaccination rates among ≧65 s (73%) and, to a lesser extent, at-risk ≤65 s (52%) in 2013/2014, over 10,000 excess deaths were reported the previous influenza season. Adult tetanus vaccines are not routinely recommended in the UK, but may be overly administered. Social influences and risk-perceptions of diseases and vaccines are known to affect vaccine uptake. We aimed to explore the socio-psychological factors that drive adult vaccination in the UK, specifically influenza and tetanus, and to evaluate whether these factors are comparable between vaccines.

**Methods:**

20 in-depth, face-to-face interviews were conducted with members of the UK public who represented a range of socio-demographic characteristics associated with vaccination uptake. We employed qualitative interviewing approaches to reach a comprehensive understanding of the factors influencing adult vaccination decisions. Thematic analysis was used to analyze the data.

**Results:**

Participants were classified according to their vaccination status as regular, intermittent and non-vaccinators for influenza, and preventative, injury-led, mixed (both preventative and injury-led) and as non-vaccinators for tetanus. We present our finding around five overarching themes: 1) perceived health and health behaviors; 2) knowledge; 3) vaccination influences; 4) disease appraisal; and 5) vaccination appraisal.

**Conclusion:**

The uptake of influenza and tetanus vaccines was largely driven by participants' risk perception of these diseases. The tetanus vaccine is perceived as safe and sufficiently tested, whereas the changing composition of the influenza vaccine is a cause of uncertainty and distrust. To maximize the public health impact of adult vaccines, policy should be better translated into high vaccination rates through evidence-based implementation approaches.

## Introduction

While immunization is one of the most effective and successful public health strategies in reducing or eliminating the health, economic and societal burden of many infectious diseases [1], major developed economies such as the US and Germany still report up to 30,000 deaths each year due to vaccine-preventable diseases, almost all in adults [2], [3]. The extraordinary success of childhood routine immunization programs across the world, which show high immunization coverage levels, has not been matched in adult programs [4]–[6]. This disparity is of increasing relevance in the context of a rapidly aging population and the attendant societal and economic burden.

Influenza and tetanus-containing boosters (tetanus boosters) are two commonly recommended vaccines for adults. Most countries follow World Health Organization (WHO) influenza vaccination recommendations: an annual vaccine, particularly for people who are at higher risk of developing influenza-related complications [7]. Although the WHO recommends an extra tetanus toxoid-containing dose in adulthood [8], recommendations for adult tetanus boosters vary across countries. For example, the US Centers for Disease Control and Prevention (CDC) recommends a 1-time dose of tetanus, diphtheria and pertussis (Tdap), followed by a tetanus and diphtheria (Td) booster every 10 years [9], whereas in France a tetanus, diphtheria and polio booster (Td/IPV) is recommended for under 25 s, a second dose at 45 years old and every 10 years for over 65 s, with one booster being replaced by a Tdap/IPV [10].

In the UK, an influenza vaccine is recommended and available free of charge for people ≧65 years old, ≤65 s with an eligible chronic health condition and pregnant women. Yet, despite comparatively high vaccination rates among ≧65 s (73%) and, to a lesser extent, ≤65 s in a clinical risk group (52%) in 2013/2014 [11], over 10,000 excess deaths were reported in UK the previous influenza season [12]. Although tetanus boosters are not included in *‘The complete routine immunisation schedule 2013/14’*, the National Health Service (NHS) recommends a Td/IPV to those who have not or have been partially immunized, or are travelling to a country with limited medical facilities [13]. The number of tetanus cases reported in the UK is low (83 in England and Wales since 2002) [14]. However, research suggests that the success of the tetanus vaccination program may be partly attributed to a mismatch between clinical practice and immunization guidelines, reflected in emergency departments' tendency to over-vaccinate patients who attend them [15]. This misalignment may also be occurring in primary care.

In countries with universal vaccination coverage, where structural barriers to access are limited, social and psychological influences such as perceived risk of diseases and vaccines are important determinants of acceptance and uptake of influenza vaccine [16]–[20]. Specifically in the UK, few qualitative studies to date have explored influenza vaccination decision-making and most have focused on the elderly [21]–[23]. Research evaluating factors driving tetanus boosters' uptake from the perspective of the vaccinee is scarce [24], [25].

To better understand social and psychological drivers of adult vaccination, we have set up a large-scale multinational qualitative study, which aims to use interview-based techniques to explore in depth adults' perceptions of vaccination and the factors that drive them to have themselves vaccinated (or not) [26]. The detailed qualitative dataset will subsequently be used to inform the development of practical survey tools that can reliably capture key determinants of vaccination behavior and predict uptake. The study that we report here is part of this larger research program. It explores the social and psychological factors that drive adult vaccination in the UK, specifically influenza and tetanus, and evaluates whether these factors are comparable between vaccines or vaccine-specific.

## Methods

This research was approved by the Imperial College Research Ethics Committee. Participants were presented with a research information sheet and briefed on confidentiality/anonymity of their data before they were asked to sign a consent form. The overall research protocol and methods used in our research program are reported in detail elsewhere [26]. We summarize them below and include specific information about the UK data collection and analysis.

### Sampling and recruitment

Interviews were carried out in three regions in the UK: London, South East and West Midlands. Although representative samples are not required in qualitative research, we sought to attain relevant perspectives by recruiting participants from areas where the majority of the UK population reside. We used a purposive sampling strategy to select 20 adult participants who were both vaccinated or not vaccinated against influenza and tetanus, and represented a range of socio-demographic characteristics associated with vaccination uptake, particularly age and health status (see Table 1). We excluded pregnant women and healthcare professionals (HCPs), as their vaccination decisions are significantly influenced by those they care for and/or regulated by healthcare authorities and professional bodies, thus some of their motivations and concerns are likely different [27], [28]. To reduce recall bias [29], only those who had been vaccinated in the past 12 months were eligible as vaccinated participants. A screening question was used to exclude participants who were fundamentally opposed to vaccination, as this stance represents only a small minority of the non-vaccinated population and thus could bias the results [30]. Participants were recruited via telephone using random dialing, sourced from telephone directories by Ipsos MORI, an international market research company.

**Table 1:** Purposive sampling strategy.

Key demographic characteristics	Min. quota
Eligible chronic condition*	**7** with
	**7** without
Gender	**8** female
	**8** male
Parent/Guardian of child/children under 18	**4** Mothers
	**4** Fathers
Age	**8** 18–49
	**4** 50–64
	**6** ≥65
Socio-economic group**	**7** ABC1
	**7** C2DE
Adults who have had one of the vaccines	**4** Influenza
	**3** Tetanus
Have had both tetanus and influenza vaccine in the last year	**6**
Have not had either vaccination in the last year	**6**
TOTAL	**20**

*These include asthma, chronic obstructive pulmonary disease or bronchitis, heart disease, kidney disease, liver disease, neurological conditions, weakened immune system due to conditions such as HIV and AIDS, or as a result of medication such as steroid tablets or chemotherapy.

**A = higher socio-economic group and E = lower socio-economic group. We used occupation and income data to determine participants' social grade.

### Piloting

The interview schedule was designed through expert consultations and a review of the relevant literature. The schedule was then tested with two researchers from Imperial College and two researchers from Ipsos MORI who were not involved in the present study. The duration and flow of the interview were discussed and the schedule was finessed as a result. The refinements to the schedule were related to wording (e.g. using ‘flu’ instead of ‘influenza’ for simplicity) and reordering and/or deletion of redundant probes. These interviews were not included in the final sample. Piloting was subsequently carried out for the first three interviews, whereby the research team observed each interview conducted by an Ipsos MORI trained interviewer behind a one-way mirror and evaluated its quality in real-time. At the end of the session, minor improvements were made to the interviewers' instructions included in the schedule.

### Procedure

Participants were interviewed face-to-face in their homes or at a central interviewing facility for approximately 60 minutes. Each participant received £70 in return for their time. Half of the interviews were conducted by an academic researcher (AW) from Imperial College and half by trained interviewer from Ipsos MORI. Two versions of the interview schedule were used: one for vaccinated and one for non-vaccinated participants (see Table 2).

**Table 2:** Interview schedule.

Interview topic (sections 1–6)	Key interview questions
1. Overview of life and values	Tell me about yourself and your life, for example, what you spend your time doing and how you enjoy yourself.
	What sorts of things do you worry about?
2. Information seeking behaviors and influences	Can you tell me how you find out what is happening generally in the world?
	And who are the people whose opinion you value or with whom you discuss important issues with? And why is that?
3. Views about health and vaccinations	Can I ask how you feel your own health is?
	When you think about your health, what are all the things that come to mind? Do you do anything to keep healthy? What sorts of things?
	Which doctors or nurses do you particularly trust and listen to, if any? And why is that? Why is that important to you?
	Thinking now about vaccinations, what are all the things that come to mind when you think about vaccinations?
	Looking at these cards, which are all adult vaccinations, please can you sort them into groups?
4. Journey to vaccination (or non-vaccination)	How would you describe to a friend how you came to have (or not to have) the vaccination? What things happened that meant you ended up getting (or not getting) vaccinated?
	What would you say happened at that point that triggered that change (or decision)? And why was that important?
	How did you know where to go for the vaccination? How did you book an appointment and fit it into your plans? What other things were competing for your time?
	Before you were vaccinated, do you remember any times when you thought about or started the process towards being vaccinated but didn't end up getting vaccinated? (vaccinated)
	Of all of those things, which would you say was the most important thing that led to you not getting vaccinated? And why is that? And the second most important thing? And the third? (non-vaccinated)
5. Children's vaccinations	In general, do you think people should vaccinate their children against tetanus? Why/why not?
	And do you think people should vaccinate their children against flu? Why/why not?
6. Factual knowledge on influenza and tetanus and related vaccines	How much would you say you know about flu/tetanus? How serious or life-threatening do you think the disease is? In general, how likely do you think you are to catch the disease?
	How much would you say you know about the vaccine for flu/tetanus? Do you happen to know how often it is recommended that you have it, or who it is recommended for?

The interview schedule comprised six sections. Section1 obtained an overview of participants' life and values, to build rapport with the interviewee and to identify important issues to assist with probing throughout the interview. Section 2 elicited participants' general information-seeking behaviors and influences. Section 3 examined participants' views toward health, HCPs and adult vaccines. Section 4 evaluated individual participants' decision making ‘journeys’ to vaccination or non-vaccination. Section 5 examined participants' attitudes toward pediatric influenza and tetanus vaccines. We aimed to understand whether or not people's views about adult vaccines correspond with their views about childhood vaccines. Finally, in section 6 we explored participants' knowledge of the two diseases and vaccines (i.e. influenza and tetanus) to understand to what extent their decision-making is influenced by factual information.

We explored the set of circumstances and emotional factors that drove participants to accept or refuse vaccination, aided by qualitative interviewing approaches aimed at obtaining information which explicit enquiry (i.e. a direct question) may fail to capture – as follows. First, throughout the interview we used an elicitation technique called *‘laddering’*, which provides a simple and systematic way of establishing people's core values and beliefs, and the linkages between these and key behaviors, in this case, vaccination [31]. In section 3, general views on adult vaccines were evaluated by asking participants to spontaneously arrange and rank, using a category of their choice, cards depicting five adult vaccines (influenza, tetanus, pneumonia, hepatitis and measles, mumps and rubella (MMR)) into one or more groups. In section 4, we employed the “Journey to vaccination” approach [26], a visual exercise in which the interviewer and the participant jointly draw a timeline that captures salient events that lead the participant to get or not to get vaccinated. These results will be presented elsewhere, as they require different analysis. Briefly, a journey to vaccination for influenza and other for tetanus is drawn for each participant. Differences and commonalities emerging from these data are identified and synthesized, and typical journeys proposed.

### Data Analysis

The recorded interviews were professionally transcribed verbatim and checked for accuracy by Ipsos MORI. To ensure reliability of coding and interpretation, all the transcripts were analyzed by the first author and 50% of the transcripts were double-coded independently by the second author [32]. Differences were resolved through discussion and review until consensus was reached. Using thematic analysis, an initial categorizing system was developed based on the study objectives and the topics explored [33], [34]. New themes and sub-themes emerging from the data analysis were identified and included when deemed relevant by the coders. Two identical thematic indexes, one for influenza and one for tetanus, were produced to code the majority of the data. Additionally, a separate matrix was developed to code and analyze the categories and rankings proposed by participants during card exercise in section 3. This coding strategy enabled us to evaluate whether the assessed factors were comparable between vaccines or were vaccine-specific. We also examined whether participants' views varied depending upon their vaccination status. The interpretation of the findings was initially carried out by the first and second author. Contributions from the rest of the authors further shaped the analysis.

## Results

### Participant characteristics

Twenty members of the UK public were interviewed in May 2013. The sample was equally split by gender. Eight participants were 18–49 yrs, seven were 50–64 yrs and five were 65+. The majority were white British (N = 16) and half were educated to university level. Just under half of the sample were retired or had a chronic health condition. Participants' characteristics are fully described in Table 3.

**Table 3:** Participant characteristics.

N	City	Vaccination status influenza	Vaccination status tetanus	Age	Gender	Chronic condition	Marital status	Children under 18	Socio-economic grade	Private health insurance	Employment status	Type of employment	Education (completed)	Religion	Ethnicity
**1**	London	Non-vaccinator	Non-vaccinator	18–49	Male	No	Married	Two	ABC1	No	Employed part-time	Supervisor	High school or equivalent	Sikh	Other ethnic group
**2**	London	Regular	Mixed	18–49	Female	Yes	Single	None	C2DE	No	Prefer not to say	Shop owner, artisan, other self-employed	Post-graduate degree	Non	White British
**3**	London	Intermittent	Injury-led	50–64	Male	No	Living with someone	One	ABC1	No	Employed full-time	Middle management	Secondary school	Prefer not to say	White British
**4**	South East	Non-vaccinator	Injury-led	50–64	Male	No	Married	One	ABC1	No	Self-employed	Business owner (full or partner)	Post-graduate degree	Hindu	White British
**5**	London	Regular	Preventative	18–49	Male	Yes	Single	None	ABC1	No	Self-employed	Shop owner, artisan, other self-employed	Post-graduate degree	Non	White British
**6**	London	Regular	Injury-led	65+	Female	No	Widowed	None	C2DE	No	Retired	Employed position, not at a desk	Primary school	Christian	White British
**7**	London	Intermittent	Injury-led	50–64	Male	No	Living with someone	One	ABC1	No	Self-employed	Business owner (full or partner)	University/college degree	Christian	White British
**8**	London	Regular	Preventative	50–64	Female	No	Married	None	ABC1	Yes	Retired	Employed position, at a desk	Secondary school	Muslim	White British
**9**	South East	Non-vaccinator	Injury-led	65+	Female	No	Divorced	None	ABC1	No	Retired	General management, top management	High school or equivalent	Muslim	White British
**10**	London	Non-vaccinator	Injury-led	18–49	Female	No	Single	None	ABC1	No	Self-employed	Shop owner, artisan, other self-employed	University/college degree	Non	White British
**11**	West Midlands	Regular	Mixed	18–49	Male	Yes	Married	Two	ABC1	No	Looking for work	Skilled manual worker	Primary school	Non	White British
**12**	West Midlands	Non-vaccinator	Non-vaccinator	50–64	Female	Yes	Single	None	ABC1	No	Retired	Employed position, at a desk	University/college degree	Buddhist	Black or Black British - Caribbean
**13**	West Midlands	Intermittent	Injury-led	50–64	Male	Yes	Married	None	ABC1	No	Retired	Middle management	University/college degree	Christian	White British
**14**	West Midlands	Non-vaccinator	Preventative	65+	Female	No	Divorced	None	C2DE	No	Retired	Business owner (full or partner)	High school or equivalent	Non	White British
**15**	London	Intermittent	Injury-led	18–49	Male	No	Single	None	C2DE	No	Self-employed	Skilled manual worker	University/college degree	Christian	Other white background
**16**	West Midlands	Regular	Non-vaccinator	18–49	Male	Yes	Living with someone	None	ABC1	No	Student	Employed position, at a desk	Primary school	Non	Other white background
**17**	West Midlands	Regular	Mixed	50–64	Male	Yes	Married	None	C2DE	No	Retired	Professional (lawyer, architect, etc.)	University/college degree	Christian	White British
**18**	West Midlands	Intermittent	Non-vaccinator	65+	Female	No	Divorced	One	C2DE	Yes	Retired	Middle management	Post-graduate degree	Christian	White British
**19**	West Midlands	Non-vaccinator	Preventative	18–49	Female	Yes	Married	None	ABC1	No	Self-employed	Other (unskilled manual worker)	High school or equivalent	Christian	White British
**20**	West Midlands	Regular	Injury-led	65+	Female	Yes	Widowed	None	C2DE	No	Retired	Employed position, not at a desk	Secondary school	Other	White British

Distinctive vaccination behavior patterns emerged from the data. Thus, the dichotomous vaccination status (i.e. vaccinated/not vaccinated) initially employed was fine-tuned; participants were classified as regular (vaccinated most or all the time; N = 8), intermittent (had vaccinated only for a period of time; N = 5) and non-vaccinators (never had vaccinated; N = 7) for influenza; and preventative (vaccinated before an injury; N = 4), injury-led (vaccinated after an injury; n = 9), mixed (both preventative and injury-led) (N = 3) and non-vaccinators (never had vaccinated; N = 4) for tetanus. In the sections that follow, we use this classification to report our findings.

### Context of vaccine perceptions

Participants widely agreed on the importance of vaccination in general and childhood vaccination in particular. When asked to rank influenza, tetanus, pneumococcal, hepatitis and MMR for adults (cards exercise), participants employed three main categories: disease severity, vaccine importance and vaccination age. Most found it difficult to categorize MMR and generally regarded it as a childhood vaccine, despite interviewers stressing that it also applies to adults.

*Severity of the disease* emerged as a key classification category. Regular vaccinators classified influenza as the most or one of the most severe diseases, followed by tetanus, pneumonia and hepatitis, although some stressed their knowledge of the latter was limited. Overall influenza non-vaccinators did not perceive this disease to be as severe as hepatitis or pneumonia, and ranked tetanus as being more severe than influenza. Those who had previously vaccinated against influenza chose *vaccine importance* to categorize vaccines. Perceived vaccine importance was related to both perceived severity and likelihood of the disease it protected against, and, consequently, the importance of “being protected”. The influenza vaccine was ranked among the two most important vaccines, followed by tetanus, pneumonia, hepatitis and MMR. Some participants used *vaccination age* as a ranking category and associated the influenza and pneumonia vaccines with adults, particularly the elderly. Hepatitis was also linked to adulthood. Tetanus was associated with teenagers and adults and MMR with children.

### Drivers and barriers to vaccination: Findings of the thematic analysis

Thematic saturation was reached at 14 interviews (i.e. no new themes appeared in the last 6 interviews that were carried out). Five overarching themes emerged from the analysis: 1) perceived health and health behaviors; 2) knowledge; 3) vaccination influences; 4) disease appraisal; and 5) vaccination appraisal. These are reported in detail below. Illustrative quotes are provided in Tables 4 and 5.

**Table 4:** Context of vaccine perceptions, perceived health and health behaviors, knowledge and influences: key emerging themes and illustrative quotes.

**Context of vaccine perceptions**
“How do vaccinations help? Well, in my opinion, they help to keep a lot of infection away. You could be infected by something and it could take your life, it could kill you if you never had that vaccination” (P20)
“…I think so many very elderly people die of flu in the winter; I think [the flu jab] is important. Yes, that one [tetanus boosters] because as we grow up, certainly teenagers and everybody onwards needs one of those… The same with that one [pneumococcal vaccine] but I think there's more flu. I'm assuming there are more flus than pneumonia around” (P14)
“I used to work in a school and I'm not aware of children getting influenza” (P8)
**Perceived health and health behaviors**
“…I'm not 20 years old you know? I get tired so quickly. By keeping fit, going to the gym, and eating healthy you know, you're putting more life into your body” (P1)
**Knowledge**
*Flu and flu vaccine*
“Well I know that people die from [flu], so it's quite serious [laughs]… I don't think that I've ever had proper flu” (P10)
“I think like the surgery where I am, I think it's diabetes, chest complaints and old age pensioners that get [the flu jab]… I believe that it is the flu virus but it's not live. And is it grown in eggs is it? Because I know my mother-in-law can't have it because she's allergic to eggs and that's where it starts. I believe that to be right. Which is why I think when people say, ‘Oh it gave me the flu’, I don't know if it does or if it doesn't, because it's never given it to me” (P11)
*Tetanus and tetanus booster*
“…tetanus? Only that I believe it can lead to lockjaw, which is quite nasty, and I also believe that it can flow through, the actual localised injury can sort of lead to, say you have it in your foot, it could lead to amputation, this is my belief” (P7)
“…I was under the awareness that it was every 10 years that you have to be vaccinated against tetanus… the influenza one in my mind is a preventative and the tetanus is also a preventative, but it can be taken after the incident” (P10)
**Vaccination influences**
*Previous salient experiences*
“I'm a bit of a chicken when it comes to [vaccines]… [As a child] I had 40 in my belly so…” (P1)
“Well, [tetanus] is quite a frightening thing to have, especially after seeing it; I didn't realise how bad it was until I saw this child. But I always knew it was quite bad because my mother was very hot on making sure you had tetanus jabs and things, yes. But yes, once it goes too far it's irreversible, lockjaw and all that” (P14)
*Family and peers*
“I've also heard various people have been ill after having the injection. I think it's meant to give you a bit of flu for you to build up antibodies or something… I thought, well, I feel well now; why should I have an anti-flu jab and then it might not make me seriously ill but it might make me feel under the weather and I don't want to feel like that” (P14)
“…. Well, it's not so much me that's frightened, my daughter, she kind of hits the roof, she's worried all the time, you see, ‘You’ve got to have it [the tetanus booster], Mum. Mum, you don't know, you don't know where the dog's been” (P6)
*Healthcare professionals and vaccine manufacturers*
“My doctor, my GP, yes, he's the one who started the ball rolling with the flu vaccine… I know when they'll start because it's on the surgery wall, you know, ‘Get your flu vaccine here’, whatever… I'm almost living in these people's pockets, you know. I bring them all panettone at Christmas” (P5)
“Yes, I fell and cut the jeans open and had a big gash… but nothing serious I thought. But I went to the doctor… she said, ‘Just when was the last time you had your tetanus jab?’ I sort of looked and thought, ‘No, I can't remember. I know I have had tetanus jabs, but…’ She said, ‘Well if you can't remember, you'll have to have a tetanus jab’, so that was that” (P7)
“Does tetanus exist in the UK or not? Don't go round to the surgery and they go, ‘You don't really need that.’ What sort of message does that give out? Yes? Do I or don't I?… Where is the provision of this information? Is it schools? Who's doing it? Is it the GP? Is it from birth? This is the thing I think they've got to worry about here…” (P5)
“…pharmaceutical companies are out to make a buck… I'm not an advocate of conspiracy theories but these people have got enormous power and a lot of money and they wouldn't be above publishing a lot of information and research that scares the hell out of us, so we all go and get a vaccine” (P8)
*Media*
“I'm friends with a professor on Facebook, and he just constantly puts things about the poison that's going into your body with the flu jab … I know he's extreme so I don't totally think, ‘Everything he says is absolutely right’, but I do think sometimes there's no smoke without fire” (19)

**Table 5:** Disease and vaccination appraisal: key emerging themes and illustrative quotes.

**Disease appraisal**
*Perceived susceptibility*
“[If] I get the flu now, I'm pretty unlikely to die from it because I'm quite healthy and I had it a couple of years ago and I was all right. So [I] think, ‘Well, it's not worth taking it now but if the danger arises, so to speak, so like as I get older, then I probably would’” (P4)
“…I know that I can't afford to get the flu because it's very, I've never had it, I know some people are in bed for a couple of weeks. If I had it, I'd probably be off for a month and it would be really bad for me. But some people don't have that attitude because maybe the risk of getting something isn't such a big deal. But I don't” (P2)
*Perceived severity*
“So, I've never, never thought of [the flu] as being a kind of dangerous thing to have… I'd seen things on the telly of people dying, that people did die from it although mostly kind of elderly and weak people” (P4)
“I suppose [I am] generally aware that [tetanus] was a dangerous thing to get and could kill you if you weren't looked after properly. Lockjaw it used to be called, didn't it?… and that was always a scary thought, a scary way to die” (P17)
*Perceived likelihood*
“I'd say 20 plus and it becomes more difficult to catch, but under that then obviously a lot of germs are being spread about. The same with older people as well, because your immune system gets a lot weaker when you're older, so it'll be easier to catch if you're old” (P16)
“A flu jab or the MMR or something, these were things that you might possibly get; you might or you might not. The tetanus, I might possibly get infected but there's more of a chance of me getting it now because I've got an injury there that's swollen (P3)
**Vaccination appraisal**
*Perceived benefits*
“So, for me, [flu vaccination] is one of these things that I fit into time… I was freelance working, so I got paid when I actually worked, so if you had flu, it's three or four weeks… you not want to be ill really because four weeks off work, not many people can afford to not be paid for four weeks” (P2)
“I thought because we travel a lot, I thought that's important to having [the tetanus boosters] up-to-date and it's not good to get…I think it's called lockjaw, isn't it, if you're not up-to-date with tetanus? That I think is very important” (P8)
*Perceived costs and practical barriers*
“A lot of people I've heard say, ‘Had my blooming flu jab and I still had flu, I was really poorly with it afterwards’. So I've heard that it's not totally effective. Whereas, as far as I'm aware, something like the measles jab, it's very, very rare to then go on to get measles after you've had your jab; with tetanus as well and so on, as long as you have it every time that you're meant to.” (P19)
“…if it's not broke, then you don't fix it, sort of thing and, okay, you can take a [flu] vaccine but I don't know what's in the vaccine. It might be fine and one would hope that it's been thoroughly researched and thoroughly tested but then I also know that there have been things in the past that have supposedly been thoroughly tested that then turn out to have something, side effect or something wrong with them. So, I won't, I don't want to take that risk unless the risk equates against the danger” (P4)
“…it was either my second or third [flu jab]… She literally just stabbed me with a needle and took me completely by surprise, so that worried me” (P16)
“It's just kind of seeing myself at risk, having the time… Just the same as having any inoculation, having to book an appointment, the accessibility to it. I don't really like needles” (P15)

### 1. Perceived health and health behaviors

Exercise, a balanced diet, not smoking and moderate alcohol consumption were perceived by most participants as desirable healthy behaviors, yet vaccination was generally not associated with the ‘healthy living’ paradigm. Those with chronic conditions were generally aware of their health status and the recommended health behaviors for preventing complications, including influenza vaccination. Accordingly, two thirds of participants who reported having a chronic health condition regularly vaccinated against influenza and had had a preventative tetanus booster. However, they were the least likely to engage in other healthy behaviors.

### 2. Knowledge

Several participants felt they did not know enough about influenza and tetanus, or the associated vaccines. Most mentioned that influenza was a potentially severe illness, yet those who had vaccinated in the past were more likely to stress it is a life-threatening disease.

Participants were widely aware about high-risk groups but were less specific about the influenza symptoms, which were commonly referred to as a “very bad cold”. Most knew the influenza vaccine was offered once a year and a third mentioned (accurately) that the vaccine did not protect against all circulating virus strands.

Tetanus was generally referred as “lockjaw” and perceived to be a very serious disease; yet additional symptoms were seldom identified. Cuts, rust and animal bites were mentioned as main sources of infection. Some believed tetanus does not exist in the UK any more. Just under half mentioned that a tetanus booster was recommended every 10 years and several noted they would get it if they had an injury, but they did not see the need to have it preventatively.

### 3. Vaccination influences

#### 3.1. Salient previous experiences

Most participants reported experiencing influenza-like symptoms once or more times over the course of their lives and several stressed, unprompted, that they knew the difference between a cold and influenza. None of the participants had contracted tetanus. Some participants who had intermittently or never vaccinated against influenza recalled a traumatic health-related experience during childhood, including painful vaccination, allergy to (injected) penicillin or frequent tonsillitis (which required penicillin injections).

#### 3.2. Family and peers

All influenza non-vaccinators, except one young healthy participant, mentioned that a family member, a friend or both had had a bad experience with the vaccine, and some recalled that although in certain seasons they had heard or read media reports about influenza-related deaths, no one they knew had been severely affected. Others, including non-vaccinators, reported that family or friends had recommended or were in favor of the influenza vaccine.

Some participants reported having had a tetanus booster due to their mother's advice or their memories of their mother's warnings about the severity of tetanus during childhood. Others mentioned advice from a family member and work colleagues' jokes about contracting tetanus as influencing their decision to seek medical help after an injury.

#### 3.3. Healthcare professionals and vaccine manufacturers

Many participants had discussed vaccinations, particularly influenza, with their general practitioner (GP). Several unvaccinated or intermittent vaccinators expressed negative feelings toward healthcare professionals, which revolved around GP's lack of empathy, NHS perverse incentives (e.g. GPs receiving payments for each administered vaccine) and general distrust of the medical profession. In contrast, all regular influenza vaccinators reported that their GPs had recommended and routinely reminded them to get vaccinated during regular consultations, through GP surgery adverts and letters. Some participants mentioned they had either got vaccinated against influenza or were reminded to do so at a pharmacy.

Receiving a tetanus booster preventatively or after an injury was generally triggered by a recommendation from a GP or an emergency department doctor. Some participants, however, reported that HCPs seemed somewhat unsure as to what the course of action was, particularly when the request for a booster was patient-led. Others mentioned they hoped their GPs would know from their electronic medical records when they were due for a tetanus booster.

Two participants, one intermittent and one non-vaccinator, raised specific concerns regarding the pharmaceutical industry's lack of trustworthiness.

#### 3.4. Media

Some participants mentioned finding out the risks of catching the influenza through the news (accessed through different mediums), whilst others had read about influenza vaccines' risks mainly through user-generated web-based sources such as personal blogs and social media.

### 4. Disease appraisal

#### 4.1. Perceived susceptibility

Susceptibility denotes constitutional vulnerability to a particular hazard, rather than the likelihood of exposure to it. All regular and some intermittent influenza vaccinators reported feeling susceptible to the disease. In contrast, although some non-vaccinators said they would consider having the vaccine when they were older and therefore more vulnerable to influenza, most felt that the risks influenza currently posed to their health did not warrant vaccination. Furthermore, some intermittent and non-vaccinators, felt they were able to prevent influenza by “improving the immune system” or “keeping healthy”.

Few participants reported feeling particularly vulnerable to tetanus, and they had had a preventative booster. Having a “good immune system” was raised by only one injury-led vaccinator as a reason why they did not feel the need to have a preventative tetanus booster.

#### 4.2. Perceived severity

Most participants acknowledged that influenza could be life-threatening for high-risk groups, but only a few were concerned about having a severe bout of the disease. The majority, particularly non-vaccinators, perceived themselves as being able to “cope” with the disease. In contrast, just under half of participants, most of whom had had a preventative booster, stated that they had a tetanus booster for fear of contracting a life-threatening disease.

#### 4.3. Perceived likelihood

Most regular influenza vaccinators felt they were likely to catch influenza, whereas some intermittent and non-vaccinators felt it was unlikely they would catch it. Participants generally interchanged susceptibility to influenza and likelihood of getting it in their interviews.

Almost half of the sample mentioned they were likely to contract tetanus, commonly due to lifestyle choices (e.g. gardening or travelling abroad), risky work environment (e.g. construction) or injuries. The other half, however, felt the likelihood of contracting tetanus was very low.

### 5. Vaccination appraisal

#### 5.1. Perceived benefits

Although many were aware that the influenza vaccine was not 100% effective, those who vaccinated regularly were more likely to acknowledge the benefits of being protected or protecting a vulnerable family member against influenza, albeit partially. Remaining productive at work/home was raised as one of the benefits of influenza vaccination. Most participants, however, were unsupportive of the pediatric influenza vaccine. Many stated that “children's immune system should be built naturally” whereas others questioned the need for an influenza vaccine for children due to perceived low prevalence of the disease among this group.

Being immunized against tetanus was considered important, yet many thought a booster was only needed after injury and not as a preventative measure. In contrast, the majority of participants felt that vaccinating children against tetanus preventatively was necessary, as they were more prone to falls and injuries.

#### 5.2. Perceived costs and practical barriers

Some intermittent and most non-vaccinators mentioned the influenza vaccine's side-effects as a main concern. The most commonly mentioned adverse effects were influenza-like symptoms and pain in the arm. Some mentioned side-effects could change yearly, depending of the composition of the vaccine, and others believed the vaccine caused influenza or was unsafe. Most regular vaccinators had not experienced memorable side-effects; those who had felt that contracting influenza was worse.

No important concerns regarding the side-effects or safety of tetanus boosters were raised. Several participants noted that the vaccine had been sufficiently tested and that they had not heard any negative things about it.

Some participants, including regular influenza vaccinators, were apprehensive about needles. Feelings of fear generally stemmed from their previous experiences or those of others. Fear of needles, however, waned when confronted with the decision of vaccinating against a disease which was perceived as a serious threat to their health: tetanus in most cases and influenza in the case of those who reported feeling particularly vulnerable to it.

Competing priorities or lack of time was raised by some as the main practical barrier to influenza vaccination. Although affordability was not an issue, two participants who were not eligible to have a free vaccine through the (publicly funded) NHS had difficulties getting it elsewhere. One reported lack of vaccine availability at the local pharmacy and the other was denied the vaccine at a supermarket due to high blood pressure. Just under a third of participants mentioned that keeping up-to-date with tetanus boosters was challenging due to their recommended time interval (10 years).

## Discussion

This study investigated the socio-psychological factors influencing adult vaccination uptake in the UK. Our results suggest that the public have no general concept of adult immunization, as they have for childhood immunization. Instead, their beliefs and attitudes are vaccine-specific and in some cases age-specific. Participants classified influenza and tetanus, and to a lesser extent pneumonia, as severe diseases. Consistent with their disease appraisal, participants felt that the influenza, tetanus and pneumococcal vaccines were important. Understandably, few participants had heard of the hepatitis vaccines or knew about hepatitis, as both hepatitis A and B are uncommon in the UK. Participants generally associated influenza and pneumonia vaccines with older age, tetanus with adolescence and MMR with childhood.

The perceived age segmentation and general lack of awareness of an adult immunization schedule may be a reflection of the way adult immunization policy in the UK has been communicated. Although a recent shift toward ‘life course vaccination’ is a move in the right direction, unlike the CDC's annually recommended adult immunization schedule [35], the NHS provides an overall schedule from 2 to 70 years old, which only includes three adult vaccines, all for over 65 s: influenza, pneumococcal and the recently introduced shingles vaccine [36]. The rest of the recommended adult vaccines fall into the “Vaccines for special groups” and “Travel vaccines” categories detailed in additional webpages, which in turn include many sub-categories and at times somewhat ambiguous exceptions. A case in point is the tetanus booster recommendation, which states: “*A tetanus vaccination is usually recommended for anyone who: has not been vaccinated before, has not been fully vaccinated, is travelling to a country with limited medical facilities, and whose last dose of the tetanus vaccine was more than 10 years ago*” [13].

Participants' knowledge about influenza and the influenza vaccine was reasonably accurate and generally acquired through mainstream media, mostly TV news, and HCPs, whereas information about vaccine risks was usually found online and in some cases in social networking sites such as Facebook. These findings resonate with those from previous research evaluating the impact of different media on vaccination behavior [37] and suggest that mass vaccination campaigns are indeed improving knowledge and prompting uptake. They also indicate that confirmation bias [38] may be driving hesitant vaccinators to seek unofficial sources that provide information at odds with scientific evidence and against vaccination – which confirms their own hesitation. Knowledge about tetanus was less specific, but most participants were aware of the severity of the disease.

Key vaccination drivers for influenza were the perceived risk of the disease, commonly assessed in the literature as a combination of perceived disease susceptibility, severity and likelihood [39], and a GP recommendation. In contrast, perceived lack of susceptibility to influenza, perceived vaccine side-effects and partial effectiveness were the main barriers to vaccination. These findings are comparable to those reported in previous studies assessing social and psychological underpinnings of vaccination [40], [41]. Whilst the drivers of tetanus vaccination were analogous to those of influenza vaccination, the barriers were somewhat different and mostly related to lack of awareness, consistent with previous research [24], [25], and variable vaccination practices. There is some indication that preventative vaccination is initiated by the vaccinee, while injury-led vaccination is instigated by HCPs.

We found that social influences played an important role in vaccination behavior, sometimes trumping participants' factual knowledge. Regular influenza vaccinators were more prone to consider the advice of relatives and peers, and have positive attitudes toward healthcare professionals than non-vaccinators. Furthermore, vaccinators reported that receiving regular reminders from their GPs about the influenza vaccine triggered vaccination uptake, which suggests that such reminders were indeed falling on fertile ground. This resonates with a vast body of literature which shows that HCP recommendation and routine reminders significantly influence vaccination uptake [42]. Conversely, participants who showed a lack of trust toward HCPs or had a relative or friend who had reported a negative experience with the vaccine were more likely to refuse vaccination or ignore their GP's recommendation. Preventative tetanus vaccinators also displayed favorable opinions toward doctors. However, some participants also felt HCPs were hesitant when asked for advice on whether or not to receive a booster, which is another indication that the current tetanus vaccination policy in the UK may lead to inconsistent or inappropriate practice [15].

A striking and novel psychological finding of this study is that previous experiences related to injections, particularly during childhood, had both a positive and a negative influence on vaccination uptake. One-third of influenza non-vaccinators reported having had a traumatic experience with vaccines, injections or medication in the past, which they stated had influenced their decision to not vaccinate against influenza. This resonates with previous research which showed that painful neonatal experiences such as circumcision or Heel Stick Capillary Blood sampling in neonates can magnify the experience of pain later in life [43], [44]. Similarly, some tetanus vaccinators recalled that the memory of their mother's warnings about the danger of contracting tetanus in childhood had influenced their decision to have a tetanus booster. These findings suggest that pain (caused by needles) and fear (of contracting a severe disease) during childhood could become both a potent vaccination deterrent or enabler. More research in this area is needed.

Consistent with previous research reporting tetanus over-vaccination in emergency departments [15], participants generally associated tetanus boosters with the treatment of injuries as opposed to the prevention of tetanus, yet, they also felt that “keeping up-to-date” with tetanus vaccination (every 10 years) was important. Our findings suggest that a convoluted tetanus immunization policy may not only underpin the routine administration of tetanus boosters in UK emergency departments but also GP practices. Excess vaccination may be further exacerbated by the recently added *‘Adult Immunisation Programme’* section of the Green Book (the official and most up-to-date source of information on immunization for HCPs in the UK), which features tetanus vaccination for adults prominently, above the influenza and pneumococcal vaccines [45]. Worryingly, our findings show that tetanus boosters may often be administered after an injury to prevent a possible infection. However, current guidelines state that the recommended course of action is administering intravenous human tetanus immunoglobulin, as the tetanus vaccine may not boost immunity in time to provide adequate protection [46].

Attitudes toward childhood vaccines were often discordant with views on the adult versions of the same vaccines. Whilst participants were accepting of children being immunized against tetanus preventatively rather than after an injury, they were largely unsupportive of pediatric influenza vaccination, consistent with parental concerns reported in previous studies [47]. Although some attitudes may be unsubstantiated (e.g. “children's immune system should be built naturally”), others, such as the lack of perceived need for pediatric influenza vaccines, may be explained by the comparatively low child mortality attributed to this disease [48]. The advantages of pediatric influenza vaccines for both the recipient and the wider community should be better communicated.

Our findings have policy implications. The UK has some of the highest seasonal influenza vaccination rates in the developed world, but gaps remain, particularly in patients with chronic conditions. National strategies to sustain and increase high influenza vaccination uptake should be built upon an evidence-based understanding of the attitudes of the public and HCPs to vaccination, and should include longitudinal evaluation of their impact [49]. A national campaign could focus on engendering feelings of susceptibility, tailored to specific at-risk population groups and their characteristics, rather than a generic fear of serious health risk, which may be inaccurate and therefore less effective for younger at risk-individuals. Communication on the risks of influenza and the benefits - and risks - of vaccination should follow risk-communication principles on how best to structure and deliver messages and be more actively diffused into social media channels. A key aspect of such communications should be introducing the concept of life course vaccination in the public's mind.

Consistent with a large body of data, we found that a GP recommendation was an important trigger for vaccination. Reminders have been effective in increasing vaccination rates in primary care practices in the UK and elsewhere, yet their utilization remains insufficient, particularly for younger at-risk individuals [50], [42]. Moreover, our findings suggest that an effective GP-led communication approach could be based on the notion of relative or contextualized risk. For example, to better understand the risks of an untrusted vaccine (e.g. influenza), it may be beneficial to draw comparisons with the risks posed by trusted vaccines (e.g. tetanus). Similarly, communicating the likelihood of experiencing side-effects from a vaccine by using the likelihood (and potential consequences) of a bout of the disease as a comparator, may facilitate evidence-based decision-making. More research on risk-communication interventions aimed at improving adult vaccination rates is warranted. Given the stated importance of negative past experiences with needles, support to help GPs to make all childhood encounters with injections as easy as possible may be a good investment in the success of vaccination programs in the future. A clearer adult tetanus immunization policy should be considered, alongside an effective dissemination plan for HCPs – as current national tetanus immunization guidelines appear not to be followed in practice.

### Limitations

This study is not without limitations. Although interviews were conducted in an open and non-judgmental manner and efforts were made to minimize availability and social desirability biases, it is possible that some participants may at times have felt compelled to give what they perceived to be rational or desirable answers. Furthermore, it is likely that recall bias may have influenced some of the participants' recollections about past experiences, particularly those around tetanus boosters. Of similar importance, our sample size, although appropriate for a qualitative study and supported by the theme saturation that was achieved, and the purposive recruitment of participants, may have an effect on the generalizability of our results. Experimental and quantitative study designs should be used to further test our findings.

## Conclusions

We found that the uptake of both influenza and tetanus vaccines is largely driven by people's risk perception of these diseases. For influenza, this appears to be mediated by trust in HCPs and the perceived risks of the vaccine, insofar the latter do not outweigh the perceived risks of influenza. The tetanus vaccine is largely viewed as sufficiently tested and safe, whereas the changing composition of the influenza vaccine is a cause of uncertainty and distrust. As we await an effective universal influenza vaccine, the advantages of newer vaccines, such as quadrivalent influenza vaccines that provide broader coverage, should be emphasized. To maximize the public health impact of current adult vaccines, policy should be better translated into high vaccination rates through evidence-based implementation approaches which draw upon a wealth of evidence from fields such as psychology and communication sciences.
