# Correction: Socio-Psychological Factors Driving Adult Vaccination: A Qualitative Study

**DOI:** 10.1371/journal.pone.0119893

**Published:** 2015-03-20

**Authors:** The PLOS ONE Staff

There are errors in the author affiliations. The affiliations should appear as shown here:

Ana Wheelock, MSc^1^; Anam Parand, PhD^1^; Bruno Rigole, MSc^2^; Angus Thomson, PhD^2^; Marisa Miraldo, PhD^3^; Charles Vincent, PhD^4^; Nick Sevdalis, PhD^1^

^1^Faculty of Medicine, Imperial College London, London, UK; ^2^Sanofi Pasteur, Lyon, France; ^3^Imperial College Business School, London, UK; ^4^Department of Experimental Psychology, University of Oxford, Oxford, UK

## References

[1] Wheelock A, Parand A, Rigole B, Thomson A, Miraldo M, Vincent C, et al (2014) Socio-Psychological Factors Driving Adult Vaccination: A Qualitative Study. PLoS ONE 9(12): e113503 doi: 10.1371/journal.pone.0113503 25490542 10.1371/journal.pone.0113503 PMC4260791

